# Long-Term Dysphagia Severity in Patients With Idiopathic Inflammatory Myopathy: A Single-Center Retrospective Study

**DOI:** 10.7759/cureus.71821

**Published:** 2024-10-18

**Authors:** Shin-Ichiro Ohmura, Keishiro Sato, Ritsu Nishimura, Toshiaki Miyamoto

**Affiliations:** 1 Rheumatology, Seirei Hamamatsu General Hospital, Hamamatsu, JPN; 2 Neurology, Seirei Hamamatsu General Hospital, Hamamatsu, JPN; 3 Rehabilitation, Seirei Hamamatsu General Hospital, Hamamatsu, JPN

**Keywords:** dysphagia, idiopathic inflammatory myopathy, long-term outcome, malignancy, survival

## Abstract

Objective

To investigate long-term dysphagia severity and survival outcomes in patients with idiopathic inflammatory myopathy (IIM).

Methods

We retrospectively included consecutive Japanese patients with IIM between April 2000 and March 2022. The primary endpoint was the complete oral intake rate according to the Food Intake LEVEL Scale (FILS) within one year after the onset of dysphagia in patients with IIM.The secondary outcome was the overall mortality rate in IIM patients with dysphagia.

Results

Of the 108 patients with IIM, 18 (16.7%) developed dysphagia during the observation period. The baseline median dysphagia severity in IIM patients with dysphagia using the FILS was 7.0 and improved to 10.0 at the final observation. Almost all IIM patients recovered from dysphagia severity, and the complete oral intake rate within one year in IIM patients with dysphagia was 72.2%. The overall mortality rate of patients with IIM patients with dysphagia was 44.4%, which is significantly lower than that of those without dysphagia (P < 0.05). Cox regression analysis demonstrated that malignancy-associated myositis was a poor prognostic factor in patients with IIM and survival outcomes in IIM patients with dysphagia were poor compared with those in patients without dysphagia when with malignancies.

Conclusions

The dysphagia severity in patients with IIM improved; however, their survival rate was lower than that of those without dysphagia when malignancy occurred.

## Introduction

Idiopathic inflammatory myopathy (IIM) is an autoimmune disease that causes inflammation of the skeletal muscles, leading to muscle weakness [1]. They are classified as dermatomyositis (DM), polymyositis (PM), and inclusion body myositis (IBM) based on their clinical, serological, and histological features [1,2]. IIM also affects muscles involved in swallowing, such as oropharyngeal, laryngeal, and esophageal muscles, which may lead to dysphagia [3]. Dysphagia development during the disease duration is very crucial because dysphagia is associated with poor prognosis and decreased quality of life due to requiring tube feeding [3-7]. The overall prevalence of dysphagia in patients with IIM is 36% globally, with IBM being the most prevalent subtype at 56% [3]. In sporadic IBM patients with dysphagia, immunosuppressive treatment does not recover their swallowing function, and dysphagia development is associated with a poor survival rate [4,7].

In contrast, in patients with PM or DM, dysphagia recovers with more aggressive treatment. The prognosis of swallowing function in DM and PM patients with dysphagia is better than that in IBM patients [8-10]. However, these data are minimal and evidence regarding the long-term dysphagia severity and survival outcomes in IIM patients with dysphagia remains unclear in Japan. Therefore, in the current study, we investigated the long-term dysphagia severity and survival outcomes in IIM patients with dysphagia in Japan. This article was previously posted to the Research Square preprint server (Preprint, Ohmura S, Sato K, Nishimura R, Miyamoto T, Improvement of Swallowing Function in Patients With Non-inclusion Body Myositis Using Food Intake LEVEL Scale; a Single-Centre Retrospective Study, 25 October 2023).

## Materials and methods

The medical records of consecutive patients with IIM who were diagnosed at the Department of Rheumatology, Seirei Hamamatsu General Hospital between April 1, 2000, and March 31, 2022, and Department of Neurology, Seirei Hamamatsu General Hospital (Hamamasu, Shizuoka, Japan) between April 1, 2000, and March 31, 2022, were reviewed in this retrospective study. In the current study, we included patients with IIM according to the probable and definite cases in the 2017 American College of Rheumatology/European League Against Rheumatism criteria for IIM [2]. Two expert rheumatologists (SO and TM) and one neurologist (KS) diagnosed IIM, particularly IBM cases. Patients aged <18 years who did not meet the classification criteria were excluded from this study. The clinical and laboratory data at diagnosis of myositis and dysphagia, initial immunosuppressive treatment, malignancy treatment, dysphagia treatment, route of nutrition administration, dietary forms during follow-up, and the cause of death were collected from the medical records. Manual muscle testing (MMT) was evaluated using a 5-point scale (1, visual or palpable contraction with no movement; 2, full range of motion (ROM) with no gravity; 3, full ROM against gravity; 4, full ROM against gravity, moderate resistance; 5, full ROM against gravity, maximal resistance). The myositis-specific autoantibody (MSA), including anti-aminoacyl-transfer RNA synthetase (ARS), including anti-Jo-1 antibody, anti-melanoma diﬀerentiation-associated gene 5 (MDA5), anti-transcription intermediary factor 1 gamma (TIF1-γ) antibody, and anti-Mi-2 antibody, were measured based on the Japanese routine clinical practice. These tests were performed using the Ouchterlony method. Malignancy-associated myositis was defined if the malignancy was diagnosed within three years of myositis onset [11-13]. All patients underwent malignancy and interstitial lung disease (ILD) screening with whole computed tomography (CT) scans. Aspiration pneumonia was defined as pneumonia occurring in patients after an aspiration episode and those with underlying dysphagia [14]. Survival, death, and the cause of death at the last observation were reviewed based on medical records.

Criteria for dysphagia　

In our hospital, physical medicine and rehabilitation determined the food choices and delivery methods based on the swallowing functions, which were evaluated using a videofluoroscopic swallowing study (VFSS) similar to a previous study [15]. All patients with dysphagia underwent swallowing evaluation repeatedly using VFSS before changing the food form. Additionally, their level of dysphagia severity was classified based on the Food Intake LEVEL Scale (FILS) [16]. In the current study, patients were diagnosed IIM with dysphagia if they fulfilled the following four conditions: 1) clinical symptoms of dysphagia; 2) abnormal findings using VFSS; 3) feeding through tube feeding, parenteral nutrition or dysphagic diet due to dysphagia; 4) no other explainable cause of dysphagia, such as stroke and Parkinson’s disease [10]. Clinical dysphagia symptoms included “food sticking in the throat,” “coughing while eating,” and “difficulty with solid foods.” There are no specific VFSS findings in IIM patients with dysphagia. Thus, based on the penetration-aspiration scale, the abnormal VFSS findings of dysphagia included pharyngeal pooling, laryngeal penetration, and aspiration [17]. Table 1 shows the FILS in this study [10,16]. The FILS score at dysphagia diagnosis and the FILS follow-up after dysphagia onset were reevaluated in the current study (SO and RN). In this study, we classified FILS scores 4-8 as non-severe, in which oral nutrition is at least partially possible, with or without the need for alternative routes of nutrition [10,16]. On the other hand, FILS scores 1-3 were classified as severe; in these patients, the nutritional routes for maintaining physical function are entirely through alternative routes other than oral intake with and without swallowing training [10,16]. Complete oral intake was defined as regaining normal function (FILS score of 9 or 10) after the onset of dysphagia, and we also evaluated the complete oral intake within one year after dysphagia onset [10,16]. FILS scores after transfer to another hospital were assessed based on medical records. Swallowing rehabilitation included tongue base retraction, effortful swallowing, Mendelsohn maneuver, falsetto, and Supraglottic swallow [18]. The interventional procedures included cricopharyngeal balloon dilation, cricopharyngeal myotomy, and gastrostomy [3].

**Table 1:** The Food Intake LEVEL Scale (FILS)

Dysphagia severity in this study	Level	Nutrition intake routes to maintain physical function	Explanation
Severe	1	Alternative nutrition, no oral intake	No swallowing training is performed except for oral care.
	2		Swallowing training not using food is performed.
	3		Swallowing training using a small quantity of food is performed.
Non-severe	4	Oral intake and alternative nutrition	Easy-to-swallow food less than the quantity of a meal (enjoyment level) is ingested orally.
	5		Easy-to-swallow food is orally ingested in one to two meals, but alternative nutrition is also given.
	6		The patient is supported primarily by ingestion of easy-to-swallow food in three meals, but alternative nutrition is used as a complement.
	7	Oral intake alone	Easy-to-swallow food is orally ingested in three meals. No alternative nutrition is given.
	8		The patient eats three meals by excluding food that is particularly difficult to swallow.
Complete oral intake	9		There is no dietary restriction, and the patient ingests three meals orally, but medical considerations are given.
	10		There is no dietary restriction, and the patient ingests three meals orally (normal).

Study outcomes

The primary endpoint was the complete oral intake rates within one year after dysphagia onset in patients with IIM. The secondary outcome was the overall survival rate in IIM patients with dysphagia after the onset of IIM.

Statistical analyses

Due to the study’s retrospective nature, we used the available number of cases and did not perform any sample size calculations and descriptive statistics of the baseline characteristics at the onset of IIM and outcomes of the patients with IIM with and without dysphagia. We used Fisher’s exact test for categorical variables to compare the two groups and the Mann-Whitney U test for continuous variables. Time-to-event analyses with survival were performed using Kaplan-Meier plots with the log-rank test. Statistical significance was set at P-values < 0.05. Furthermore, to identify risk factors associated with mortality, we constructed Cox regression hazard models using death as the event and risk factors previously reported as predictors, age at diagnosis, dysphagia, and malignancy [19]. All statistical analyses were performed using EZR software (Saitama Medical Center, Jichi Medical University, Saitama, Japan), which is a graphical user interface for R (The R Foundation for Statistical Computing, Vienna, Austria) [20].

Ethics

This study was approved by the Seirei Hamamatsu General Hospital ethics review committee (number: 4232) and conducted according to the Declaration of Helsinki and the 2017 Ethical Guidelines for Medical and Health Research Involving Human Subjects in Japan. Written informed consent was waived due to the study’s retrospective design, and information on the right to opt out of the study was presented by the Seirei Hamamatsu General Hospital ethics review committee (number: 4232).

## Results

Clinical characteristics, treatments, and outcomes of IIM patients with and without dysphagia

**Figure 1:**
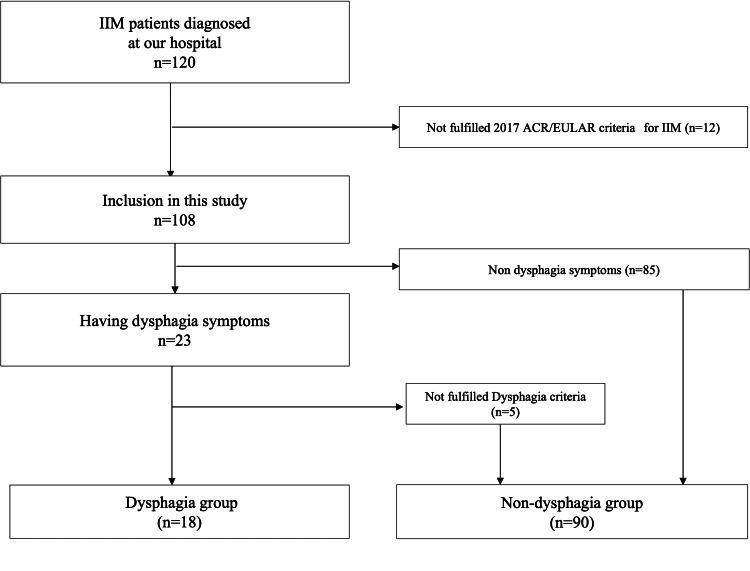
Flowchart of patient inclusion IIM: idiopathic inflammatory myopathy; ACR: American College of Rheumatology; EULAR: European league against rheumatism.

All 108 patients were Japanese, of which 18 (16.7%) were in the dysphagia group, and 90 (83.3%) were in the non-dysphagia group (Figure 1).

Fifteen (83.3%) patients developed dysphagia at the first IIM diagnosis, all of whom were DM and PM. Table 2 shows the baseline demographic and clinical characteristics, treatment, and death during observation.

**Table 2:** Baseline demographic and clinical characteristics, and treatments of IIM patients with and without dysphagia. *P-values of < 0.05 is significant; †According to the 2018 National Health and Nutrition Survey in Japan, the percentage with a body mass index >25.0 is 32.0% in men and 21.9% in women; ‡Purine synthesis inhibitors include azathioprine, mycophenolate mofetil, and mizoribine; DM: dermatomyositis; PM: polymyositis; IMNM: immune-mediated necrotizing myopathy; IBM: Inclusion body myositis; IIM: idiopathic inflammatory myopathy; MMT: manual muscle testing; anti-ARS: anti-aminoacyl-transfer RNA synthetase; anti-MDA5: anti-melanoma diﬀerentiation-associated gene 5; anti-TIF1-γ: anti-transcription intermediary factor 1-γ; CTDs: connective tissue diseases.

Clinical characteristics	Total (n=108)	Dysphagia group (n=18)	Non-dysphagia group (n=90)	P-value
Definite/probable	77/31	12/6	65/25	0.78
Female gender (n, %)	74 (68.5)	13 (72.2)	61 (67.8)	0.79
Age at diagnosis (years old)	57.5 (20.4, 86.8)	69.5 (50.4, 81.3)	55.4 (20.4, 86.8)	<0.001*
Body mass index†	21.2 (14.4, 36.7)	21.7 (17.1, 33.3)	21.1 (14.4, 36.7)	0.51
From onset to diagnosis of IIM (day)	84.5 (2.0, 5921.0)	84.5 (2.0, 3839.0)	84.0 (2.0, 5921.0)	0.95
DM (n, %)	71 (65.7)	9 (50.0)	62 (68.9)	0.17
PM (n,%)	25 (23.1)	4 (22.2)	21 (23.3)	1.0
IMNM (n,%)	6 (5.6)	2 (11.1)	4 (4.4)	1.0
IBM (n,%)	6 (5.6)	3 (16.7)	3 (3.3)	0.06
Malignancy-associated myositis (n,%)	20 (18.5)	12 (66.7)	8 (8.9)	<0.001*
Interstitial lung disease (n, %)	60 (55.6)	3 (16.7)	57 (63.3)	<0.001*
Overlap with the other CTDs (n, %)	14 (13.0)	1 (5.6)	13 (14.4)	0.46
Fever (>37.5°C) (n, %)	34 (31.5)	1 (5.6)	33 (36.7)	0.01*
Arthralgia/arthritis (n, %)	50 (46.3)	4 (22.2)	46 (51.1)	0.04*
Myalgia/muscle weakness (n, %)	98 (90.7)	17 (94.4)	81 (90.0)	1.0
Proximal upper limb MMT (0-5)	4.0 [2.0, 5.0]	3.5 [2.0, 5.0]	4.0 [2.0, 5.0]	0.003*
Proximal lower limb MMT (0-5)	4.0 [2.0, 5.0]	3.0 [2.0, 4.0]	4.0 [2.0, 5.0]	<0.001*
White blood cell count (×10^3^/μL)	6645 (3040, 21250)	6945 (4240, 11270)	6630 (3040, 21250)	0.78
Albumin (g/dL)	3.30 [1.90, 4.60]	3.05 [1.90, 4.10]	3.35 [2.10, 4.60]	0.03*
Aspartate aminotransferase (U/L)	84.0 [16.0, 535.0]	111.0 [16.0, 346.0]	81.0 [17.0, 535.0]	0.50
*Lactate dehydrogenase (U/L)*	511 (182, 2245)	476 (192, 1430)	515 (182, 2245)	0.90
Creatine kinase (U/L)	1466 (29, 13207)	1646 (123, 11274)	1466 (29, 13207)	0.97
Aldolase (U/L)	21.0 [3.3, 196.8]	15.4 [8.5, 70.0]	24.05 [3.30, 196.80]	0.17
C-reactive protein (mg/dL)	0.44 [0.00, 12.70]	0.43 [0.10, 12.70]	0.44 [0.00, 10.40]	0.15
Anti-ARS positive (n, %)	30/76 (39.5)	0/10 (0.0)	30/66 (45.5)	0.005*
Anti-Jo-1 positive (n, %)	17/108 (15.7)	0/18 (0.0)	17/90 (18.9)	0.07
Anti-ARS positive but anti-Jo-1 negative (n, %)	13/76 (17.1)	0/10 (0.0)	13/66 (19.7)	0.20
Anti-TIF1-γ positive (n, %)	5/62 (8.0)	2/6 (33.3)	3/56 (5.4)	0.07
Anti-Mi-2 positive (n, %)	3/61 (4.9)	0/5 (0.0)	3/56 (5.4)	1.0
Anti-MDA5 positive (n, %)	3/60 (5.0)	0/5 (0.0)	3/55 (5.5)	1.0
Glucocorticoid (n,%)	101 (93.5)	14 (77.8)	87 (96.7)	0.01*
Prednisolone (mg/day)	50.0 [0.0, 100.0]	50.0 [0.0, 80.0]	50.0 [0.0, 100.0]	0.23
Glucocorticoid pulse therapy (n,%)	70 (64.8)	10 (55.6)	60 (66.7)	0.42
Immunosuppressive agents (n,%)	63 (58.3)	6 (33.3)	57 (63.3)	0.03*
Cyclophosphamide (n,%)	10 (9.3)	1 (5.6)	9 (10.0)	1.0
Methotrexate (n,%)	12 (11.1)	3 (16.7)	9 (10.0)	0.42
Calcineurin inhibitors (n,%)	41 (38.0)	2 (11.1)	39 (43.3)	0.02*
Purine synthesis inhibitors‡(n,%)	6 (5.6)	1 (5.6)	5 (5.6)	1.0
Intravenous immunoglobulin (n,%)	10 (9.3)	6 (33.3)	4 (4.4)	0.001*
Treatment for malignancy (n,%)	16 (14.8)	9 (50.0)	7 (7.8)	<0.001*
Surgical treatment (n,%)	13 (12.0)	6 (33.3)	7 (7.8)	0.008*
Chemotherapy (n,%)	10 (9.3)	6 (33.3)	4 (4.4)	0.001*
Radiation therapy (n,%)	3 (2.8)	2 (11.1)	1 (1.1)	0.07
Death	31 (28.7)	10 (55.6)	21 (23.3)	0.01*
Malignancies (n,%)	13 (12.0)	8 (44.4)	5 (5.6)	<0.001*
Infectious diseases (n,%)	5 (4.6)	2 (11.1)	3 (3.3)	0.19
Interstitial lung disease (n,%)	4 (3.7)	0 (0.0)	4 (4.4)	1.0
Cardiovascular diseases (n,%)	4 (3.7)	0 (0.0)	4 (4.4)	1.0
Others (n,%)	5 (4.6)	0 (0.0)	5 (5.6)	0.59
Observation period (year)	5.06 [0.13, 22.61]	2.61 [0.70, 10.47]	5.48 [0.13, 22.61]	0.02*

Seventy-one patients (65.7%) had DM, and 17 (15.7%) of whom had anti-ARS antibodies, including eight cases positive for anti-Jo-1 antibodies and nine cases positive for anti-ARS antibodies other than the Jo-1 antibodies. There were no patients positive for anti-ARS antibodies in the dysphagia group.

The patients in the dysphagia group were significantly older at diagnosis and had a higher proportion of malignancy-associated myositis than those in the non-dysphagia group. On the other hand, ILD, fever, arthralgia/arthritis, MMT, serum albumin levels, and anti-ARS antibody positivity were significantly lower in the Dysphagia group than in the Non-dysphagia group. In VFSS findings in the dysphagia group, pharyngeal pooling was observed in 18 (100%) patients, laryngeal penetration in 14(77.8%), and aspiration in 5(27.8%).

**Figure 2:**
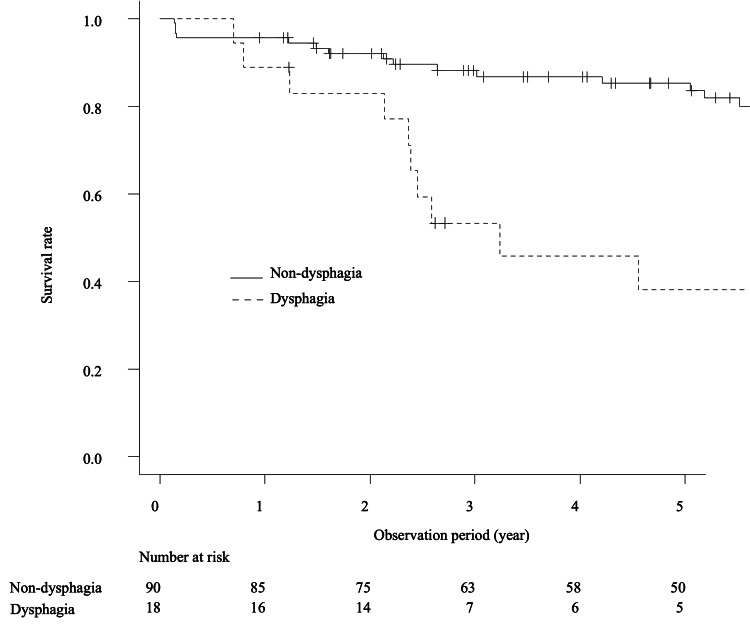
Survival rate in IIM patients with and without dysphagia IIM: idiopathic inflammatory myopathy.

The proportion of patients with IBM was higher in the dysphagia group than in the non-dysphagia group (P = 0.06). Regarding treatment, intravenous immunoglobulin (IVIG) and cancer treatment frequency were significantly higher in the dysphagia group, whereas that of glucocorticoid and immunosuppressive agents was significantly lower than in the non-dysphagia group. Ten (55.6%) and 21 (23.3%) patients died in the dysphagia and non-dysphagia groups, respectively. The dysphagia group had a significantly higher mortality and death from malignancy rate than the non-dysphagia group (P < 0.001). However, no patient with dysphagia died from ILD in the dysphagia group. Kaplan-Meier curves demonstrated that the five-year survival rates in the dysphagia group and non-dysphagia group were 38.1% [95% confidence interval (CI): 15.2-61.0%} and 85.3% (95% CI: 75.4-91.4%), respectively (P < 0.001) (Figure 2).

Clinical characteristics, treatments, and outcomes of IIM patients with dysphagia diagnosis at dysphagia graded as dysphagia severity using FILS are shown in Table 3. The complete oral intake rate within one year was similar between the groups.

**Table 3:** Baseline demographic and clinical characteristics, and treatments in IIM patients at the diagnosis of dysphagia which was graded as dysphagia severity by the FILS Data are presented as number (%) or median [range]. Severe dysphagia was defined as FILS level 1-3 and non-severe dysphagia was defined as FILS level 4-8. *P values of <0.05 is significant. †According to the 2018 National Health and Nutrition Survey in Japan, the percentage with a body mass index of more than 25.0 is 32.0% in Men and 21.9% in Females. †Purine synthesis inhibitors include azathioprine, mycophenolate mofetil, and mizoribine. ‡Complete oral intake was defined as FILS level 9-10. DM: dermatomyositis; PM: polymyositis; IMNM: immune-mediated necrotizing myopathy; IBM: Inclusion body myositis; IIM: idiopathic inflammatory myopathy; MMT: manual muscle testing; anti-ARS: anti-aminoacyl-transfer RNA synthetase; anti-MDA5: anti-melanoma diﬀerentiation-associated gene 5; anti-TIF1-γ: anti-transcription intermediary factor 1-γ; CTDs: connective tissue diseases.

Clinical characteristics	Total (n=18)	Severe dysphagia group (n=6)	non-Severe dysphagia group (n=12)	P-value
Diagnosis of IIM (Definite/probable)	12/6	4/2	8/4	1.0
Female gender (n,%)	13 (72.2)	5 (83.3)	8 (66.7)	0.62
Age at diagnosis (years old)	69.5 [50.4, 81.3]	68.6 [50.9, 74.0]	70.6 [50.4, 81.3]	0.30
Age diagnosis of dysphagia (years old)	69.5 [50.4, 81.2]	68.6 [50.9, 73.9]	70.5 [50.4, 81.2]	0.26
Body mass index*	21.7 [17.1, 33.3]	21.1 [17.6, 23.9]	22.5 [17.1, 33.3]	0.40
From symptoms onset to IIM diagnosis (day)	84.5 [2.0, 3839.0]	75.5 [23.0, 118.0]	95.5 [2.0, 3839.0]	0.48
From dysphagia onset to IIM diagnosis (day)	9.5 (-70, 986)	15.5 [-70.0, 32.0]	6.5 [-55.0, 986.0]	0.67
DM (n, %)	9 (50.0)	4 (66.7)	5 (41.7)	0.62
PM (n,%)	4 (22.2)	2 ( 33.3)	2 (16.7)	0.57
IMNM (n,%)	2 (11.1)	0 ( 0.0)	2 (16.7)	0.53
IBM (n,%)	3 (16.7)	0 (0.0)	3 (25.0)	0.52
Malignancy-associated myositis (n,%)	10 (55.6)	3 (50.0)	7 (58.3)	1.0
Interstitial lung disease (n,%)	3 (16.7)	2 (33.3)	1 (8.3)	0.25
Overlap with the other CTDs (n,%)	1 (5.6)	0 (0.0)	1 (8.3)	1.0
Fever (>37.5°C) (n,%)	1 (5.6)	1 (16.7)	0 (0.0)	0.33
Arthralgia/arthritis (n,%)	4 (22.2)	2 (33.3)	2 (16.7)	0.57
Myalgia/muscle weakness (n,%)	17 (94.4)	5 (83.3)	12 (100.0)	0.33
Proximal upper limb MMT (0-5)	3.5 [2.0, 5.0]	3.0 [2.0, 5.0]	4.0 [2.0, 5.0]	0.56
Proximal lower limb MMT (0-5)	3.0 [2.0, 4.0]	3.0 [2.0, 4.0]	3.0 [2.0, 4.0]	1.0
White blood cell count (/μL)	6945 (4240, 11270)	6515 (4520, 11270)	7445 (4240, 10100)	0.51
Albumin (g/dL)	3.05 [1.90, 4.10]	2.60 [2.00, 3.40]	3.10 [1.90, 4.10]	0.09
Aspartate aminotransferase (U/L)	111(16, 346)	121 (44, 188)	95 (16, 346)	0.78
Lactate dehydrogenase (U/L)	476 (192, 1430)	533 (337, 679)	475 (192, 1430)	0.71
Creatine kinase (U/L)	1646 (123,11274)	1605 (123,6835)	1646 (178,11274)	0.93
Aldolase (U/L)	15.4 [8.5, 70.0]	15.4 [8.5, 21.1]	15.4 [10.3, 70.0]	0.81
C-reactive protein (mg/dL)	0.43 [0.10, 12.70]	1.80 [0.10, 2.90]	0.40 [0.10, 12.70]	0.71
Anti-ARS positive (n,%)	0/10 (10.0)	0/3 (0.0)	0/7 (14.3)	1.0
Anti-TIF1-γ positive (n,%)	2/6 (33.3)	0/2 (0.0)	2/4 (50.0)	0.47
Anti-Mi-2 positive (n,%)	0/5 (0.0)	0/2 (0.0)	0/3 (0.0)	1.0
Anti-MDA5 positive (n,%)	0/5 (0.0)	0/2 (0.0)	0/3 (0.0)	1.0
Glucocorticoid (n,%)	14 (77.8)	6 (100.0)	8 (66.7)	0.25
Prednisolone (mg/day)	50.0 [0.0, 80.0]	50.0 [50.0, 80.0]	50.0 [0.0, 60.0]	0.73
Glucocorticoid pulse therapy (n,%)	10 (55.6)	5 (83.3)	5 (41.7)	0.15
Immunosuppressive agents (n,%)	6 (33.3)	3 (50.0)	3 (25.0)	0.34
Cyclophosphamide (n,%)	1 (5.6)	1 (16.7)	0 (0.0)	0.33
Methotrexate (n,%)	3 (16.7)	1 (16.7)	2 (16.7)	1.0
Calcineurin inhibitors (n,%)	2 (11.1)	1 (16.7)	1 (8.3)	1.0
Purine synthesis inhibitors (n,%)†	1 (5.6)	0 (0.0)	1 (8.3)	1.0
Intravenous immunoglobulin (n,%)	6 (33.3)	3 (50.0)	3 (25.0)	0.34
Treatment for malignancy (n,%)	9 (50.0)	3 (50.0)	6 (50.0)	1.0
Surgical treatment (n,%)	6 (33.3)	3 (50.0)	3 (25.0)	0.34
Chemotherapy (n,%)	6 (33.3)	3 (50.0)	3 (25.0)	0.34
Swallowing rehabilitation (n,%)	18 (100.0)	6 (100.0)	12 (100.0)	1.00
Tube feeding/parenteral nutrition (n,%)	8 (44.4)	6 (100.0)	2 (16.7)	0.002*
Interventional procedure for dysphagia (n,%)	14 (77.8)	6 (100.0)	8 (66.7)	0.25
Cricopharyngeal balloon dilatation (n,%)	14 (77.8)	6 (100.0)	8 (66.7)	0.25
Cricopharyngeal myotomy (n,%)	1 (5.6)	1 (16.7)	0 (0.0)	0.33
Gastrostomy therapy (n,%)	2 (11.1)	0 (0.0)	2 (16.7)	0.53
FILS at the diagnosis of dysphagia	7.0 [2.0, 8.0]	2.0 [2.0, 3.0]	7.0 [7.0, 8.0]	<0.001*
FILS at 1-year follow up	10.0 [7.0, 10.0]	9.0 [7.0, 10.0]	10.0 [6.0, 10.0]	0.60
FILS at the last follow up	10.0 [1.0, 10.0]	9.0 [7.0, 10.0]	10.0 [1.0, 10.0]	0.79
Complete oral intake within 1-year^‡^ (n,%)	13 (72.2)	4 (66.7)	9 (75.0)	1.0
Aspiration pneumonia during observation (n,%)	6 (33.3)	3 (50.0)	3 (25.0)	0.34
Relapse of dysphagia (n,%)	2 (11.1)	1 (16.7)	1 (8.3)	1.0
Death	10 (55.6)	4 (66.7)	6 (50.0)	0.64
Malignancies (n,%)	8 (44.4)	2 (33.3)	6 (50.0)	0.64
Infectious diseases (n,%)	2 (11.1)	2 (33.3)	0 (0.0)	0.10
Interstitial lung disease (n,%)	0 (0.0)	0 (0.0)	0 (0.0)	1.0
Cardiovascular diseases (n,%)	0 (0.0)	0 (0.0)	0 (0.0)	1.0
Others (n,%)	0 (0.0)	0 (0.0)	0 (0.0)	1.0
Observation period (year)	2.61 [0.70, 10.47]	2.42 [0.70, 10.47]	2.67 [1.23, 9.46]	0.57
Observation period after dysphagia onset (year)	2.47 [0.6, 10.46]	2.31 [0.6, 10.46]	2.56 [1.13, 5.47]	0.71

Clinical characteristics, treatments, and outcomes in IIM patients with dysphagia are classified based on the immune-mediated necrotizing myositis, DM, and IBM subtypes.

Table 4 shows the clinical characteristics, treatments, and outcomes of IIM patients with dysphagia classified based on the immune-mediated necrotizing myositis (IMMN), DM, and IBM subtypes.

**Table 4:** Baseline demographic and clinical characteristics and treatments in patients with IIM and dysphagia classified based on the IMMN, DM, and IBM subtypes Data are presented as number (%) or median (range). *According to the 2018 National Health and Nutrition Survey in Japan, the percentage with a body mass index >25.0 is 32.0% in men and 21.9% in women. †Purine synthesis inhibitors include azathioprine, mycophenolate mofetil, and mizoribine. ‡Complete oral intake was defined as FILS level 9-10. DM: dermatomyositis; PM: polymyositis; IMNM: immune-mediated necrotizing myopathy; IBM: Inclusion body myositis; IIM: idiopathic inflammatory myopathy; MMT: manual muscle testing; anti-ARS: anti-aminoacyl-transfer RNA synthetase; anti-MDA5: anti-melanoma diﬀerentiation-associated gene 5; anti-TIF1-γ: anti-transcription intermediary factor 1-γ; CTDs: connective tissue diseases.

Clinical characteristics	IMNM group (n=2)	DM group (n=9)	IBM group (n=3)
Diagnosis of IIM (Definite/probable)	0/2	9/0	3/0
Female gender (n,%)	1 (50.0)	7 (77.8)	1 (33.3)
Age at diagnosis (years old)	72.8 [65.7, 79.9]	69.4 [50.4, 81.3]	74.0 [72.5, 78.8]
Age diagnosis of dysphagia (years old)	72.7 [655, 80.0]	69.5 [50.4, 81.2]	76.9 [76.3, 78.8]
Body mass index*	25.2 [17.1, 33.3]	21.6 [17.6, 26.4]	23.4 [20.8, 23.8]
From symptoms onset to IIM diagnosis (day)	95.5 [84.0, 107.0]	64.0 [2.0, 138.0]	1835.0 [1358.0, 3839.0]
From dysphagia onset to IIM diagnosis (day)	16.5 [0.0, 33.0]	4.0 [-51.0, 32.0]	953.0 [626.0, 986.0]
Malignancy-associated myositis (n,%)	2 (100.0)	7 (77.8)	0 (0.0)
Interstitial lung disease (n,%)	0 (0.0)	3 (33.3)	0 (0.0)
Overlap with the other CTDs (n,%)	0 (0.0)	0 (0.0)	0 (0.0)
Fever (>37.5°C) (n,%)	0 (0.0)	1 (11.1)	0 (0.0)
Arthralgia/arthritis (n,%)	0 (0.0)	3 (33.3)	0 (0.0)
Myalgia/muscle weakness (n,%)	2 (100.0)	8 (88.9)	3 (100.0)
Proximal upper limb MMT (0-5)	3.0 [2.0, 4.0]	3.0 [2.0, 5.0]	4.0 [4.0, 4.0]
Proximal lower limb MMT (0-5)	3.0 [2.0, 4.0]	4.0 [2.0, 4.0]	2.0 [2.0, 3.0]
White blood cell count (/μL)	6105 (4240, 7970)	6630 (4520, 9050)	6640 (5760, 7640)
Albumin (g/dL)	3.25 [3.10, 3.40]	3.00 [2.00, 3.60]	3.80 [3.70, 4.10]
Aspartate aminotransferase (U/L)	255.5 [165.0, 346.0]	82.0 [44.0, 331.0]	26.0 [16.0, 46.0]
Lactate dehydrogenase (U/L)	1107.0 [784.0, 1430.0]	438.0 [337.0, 859.0]	201.0 [192.0, 516.0]
Creatine kinase (U/L)	7515.5 [3757.0, 11274.0]	1056.0 [123.0, 9927.0]	197.0 [178.0, 1196.0]
Aldolase (U/L)	8.35 [46.70, 70.00]	12.50 [8.50, 21.10]	15.40 [15.40, 15.40]
C-reactive protein (mg/dL)	0.29 [0.17, 0.40]	0.40 [0.10, 2.40]	2.40 [0.20, 8.59]
Glucocorticoid (n,%)	2 (100.0)	8 (88.9)	0 (0.0)
Prednisolone (mg/day)	55.0 [50.0, 60.0]	50.0 [0.0, 80.0]	0.0 [0.0, 0.0]
Glucocorticoid pulse therapy (n,%)	1 (50.0)	7 (77.8)	0 (0.0)
Immunosuppressive agents (n,%)	1 (50.0)	3 (33.3)	0 (0.0)
Cyclophosphamide (n,%)	0 (0.0)	1 (11.1)	0 (0.0)
Methotrexate (n,%)	1 (50.0)	0 (0.0)	0 (0.0)
Calcineurin inhibitors (n,%)	1 (50.0)	1 (11.1)	0 (0.0)
Purine synthesis inhibitors (n,%)†	0 ( 0.0)	1 (11.1)	0 (0.0)
Intravenous immunoglobulin (n,%)	1 (50.0)	4 (44.4)	0 (0.0)
Treatment for malignancy (n,%)	1 (50.0)	5 (55.6)	0 (0.0)
Surgical treatment (n,%)	1 (50.0)	3 (33.3)	0 (0.0)
Chemotherapy (n,%)	0 (0.0)	4 (44.4)	0 (0.0)
Swallowing rehabilitation (n,%)	2 (100.0)	9 (100.0)	3 (100.0)
Tube feeding/parenteral nutrition (n,%)	0 (0.0)	4 (44.4)	2 (66.7)
Interventional procedure for dysphagia (n,%)	0 (0.0)	9 (100.0)	2 (66.7)
Cricopharyngeal balloon dilatation (n,%)	0 (0.0)	9 (100.0)	2 (66.7)
Cricopharyngeal myotomy (n,%)	0 (0.0)	1 (11.1)	0 (0.0)
FILS at the diagnosis of dysphagia	7.0 [7.0, 7.0]	7.0 [2.0, 8.0]	7.0 [7.0, 8.0]
FILS at the last follow up	10.0 [10.0, 10.0]	10.0 [7.0, 10.0]	1.0 [1.0, 7.0]
Complete oral intake within 1-year^‡^ (n,%)	2 (100.0)	7 (77.8)	0 (0.0)
Gastrostomy therapy (n,%)	0 (0.0)	0 ( 0.0)	2 (66.7)
Aspiration pneumonia during observation (n,%)	0 (0.0)	3 (33.3)	3 (100.0)
Relapse of dysphagia (n,%)	0 (0.0)	1 (11.1)	0 (0.0)
Death	1 (50.0)	7 (77.8)	0 (0.0)
Malignancies (n,%)	1 (50.0)	5 (55.6)	0 (0.0)
Infectious diseases (n,%)	0 (0.0)	2 (22.2)	0 (0.0)
Interstitial lung disease (n,%)	0 (0.0)	0 (0.0)	0 (0.0)
Cardiovascular diseases (n,%)	0 (0.0)	0 (0.0)	0 (0.0)
Others (n,%)	0 (0.0)	0 (0.0)	0 (0.0)
Observation period (year)	2.90 [1.23, 4.57]	2.39 [0.60, 10.46]	7.48 [7.36, 9.46]
Observation period after dysphagia onset (year)	2.85 [1.13, 4.58]	2.39 [0.70, 10.47]	3.69 [1.94, 5.47]

No patient with IBM received any immunosuppressive treatments. The complete oral intake rate in the DM and IMMN groups was higher than that in the IBM group, and two patients with IBM eventually underwent gastrostomies. However, the survival rate in the IBM group was higher than that in the DM and IMMN groups.

Factors associated with death in patients with IIM

Cox regression analysis revealed that dysphagia is not a poor prognostic factor but malignancy-associated myositis was a poor prognostic factor in patients with IIM (Table 5).

**Table 5:** Multivariable Cox proportional hazard model for death in IIM patients CI: confidence interval; HR: hazard ratio; IIM: idiopathic inflammatory myopathy. *P-values of <0.05 is significant.

Variables	HR	[95%CI]	P-value
Age at diagnosis of IIM	1.02	[0.99 to 1.05]	0.22
Malignancy-associated myositis	5.78	[2.59 to 12.89]	<0.001*
Dysphagia	1.50	[0.59 to 3.79]	0.39

## Discussion

In this single-center retrospective study, we investigated the long-term dysphagia severity and survival outcomes in IIM patients with dysphagia and showed the following two points: First, dysphagia severity in almost all patients with IIM improved immunosuppressive and swallowing rehabilitation treatments. Second, the prognosis for IIM patients with dysphagia was poor, mainly leading to malignancies.

In the current study, although all non-IBM patients with dysphagia improved their dysphagia severity, almost all patients with IBM eventually required gastrostomy.

Previous reports have shown that almost all non-IBM patients with dysphagia improved their swallowing function and 73.1% recovered their complete oral intake within one year [6.10]. In addition, 82.2-90.0% of patients with DM or PM with dysphagia achieved complete oral intake with immunosuppressive therapy and IVIG treatment [8,9]. Notably, previous studies showed that IVIG treatment was effective for IIM patients with dysphagia [8,9]. In this study, the complete oral intake within one year rate in non-IBM patients treated with IVIG was higher than that in patients not treated with IVIG; however, this difference was not significant (100% vs. 77.8%; P=0.49). In addition, there were no significant differences between non-IBM patients treated with and without immunosuppressive treatments (83.3% vs. 88.9%; P=1.0). In this study, all non-IBM patients did swallowing rehabilitation and 12 patients (80%) of them underwent interventional procedures. Thus, it is possible that not only immunosuppressive treatments but also interventional procedures could influence both swallowing function outcomes.

On the other hand, patients with IBM did not respond to immunosuppressive treatments, and no treatment slowed the disease progression [21]. In the current study, disease progression occurred in all patients with IBM, and they did not receive any immunosuppressive treatment when they had dysphagia. In addition, previous reports have shown that the swallowing function outcomes of IBM patients with dysphagia were very poor compared with those without IBM, and IBM patients with dysphagia did not recover their swallowing function with immunosuppressive therapy, and their gastrostomy rate was 24%, which was consistent with our study [3-7].

In addition, several investigators have reported the effectiveness of interventional procedures for IBM patients with dysphagia [4,5,22]. In the current study, two IBM patients received interventional procedures, specifically cricopharyngeal balloon dilation (n = 2), but the treatment was ineffective. Previous studies showed that cricopharyngeal balloon dilation can improve dysphagia symptoms [4,5,22]; however, the effect may not be permanent, and repeated procedures are required. On the other hand, one study showed that cricopharyngeal myotomy was effective in IBM patients with dysphagia [5]. In the current study, no patient with IBM received cricopharyngeal myotomy, and it is possible that physicians should have considered cricopharyngeal myotomy for IBM patients with dysphagia.

The survival rates in IIM patients with dysphagia in the current study were significantly lower than those of patients without dysphagia. Previous studies have also shown that IIM patients with dysphagia had poor outcomes compared with those without dysphagia due to the high malignancy risk. Notably, non-IBM patients with dysphagia have a higher prevalence of malignancies and poor prognosis than those with IBM [4, 11, 23-26].

**Figure 3:**
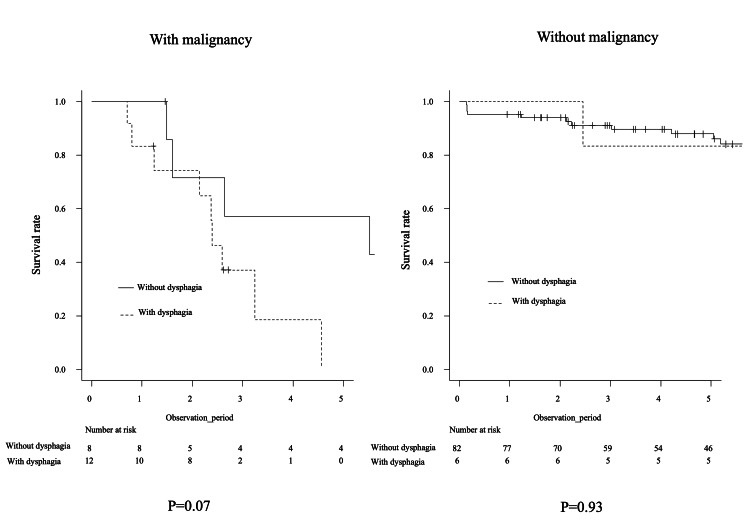
Survival rate in IIM patients with and without malignancy IIM: idiopathic inflammatory myopathy.

In the current study, eight patients (44.4%) in the Dysphagia group died due to malignancies. Five of these patients had DM and one was anti-TIF1-γ antibody positive. A previous study showed that DM patients with anti-TIF1-γ positivity and dysphagia frequently developed malignancy [27]. On the other hand, a previous study showed that IIM patients with and without malignancy differ in status and separate analyses should be considered when analyzing mortality rates [26]. Dysphagia is not associated with increased mortality in patients with IIM without cancer according to the study [26]. In the current study, survival outcomes in IIM patients with dysphagia were not poor compared with those in patients without dysphagia and without malignancies (Figure 3), which was consistent with a previous study [26].

Our findings suggest that physicians should encourage IIM patients not to be pessimistic since almost all patients with IIM may experience improved swallowing functions through immunosuppressive treatment, swallowing rehabilitation, nutritional therapy, and a multidisciplinary approach. In addition, patients with IIM may not have poor outcomes if malignancy does not occur or is treated in the early stage. Therefore, IIM patients with dysphagia will have no obstacles attributed to this disease that can hinder them from enjoying their lives.

This study has some limitations owing to its design and sample size. Since IIM is a very rare disease, previous studies on IIM with dysphagia included a very small sample size [4-8]. In the current study, we included only 18 patients with IIM and dysphagia, particularly three IBM patients with dysphagia, which may have limited the generalizability of the findings. Due to the relatively small number of patients, the study might not have had sufficient sensitivity or accuracy to fully capture the impact of dysphagia on survival. Therefore, future studies with larger cohorts are required to confirm these findings. Another limitation is that we could not obtain MSA in all IIM patients. MSA is associated with clinical phenotype and outcome in patients with IIM. However, MSA has been measured since 2017 in Japan and our study might have missed many MSA values because of its retrospective design. In the current study, 76 (70.4%) of the 108 patients with IIM measured at least one MSA (anti-ARS, anti-MDA5, anti-TIF1-γ, and anti-Mi-2), with 41 (53.9%) of them being positive. Among these limited patients, the positive rate in dysphagic cases was 8/10 (80.0%), which was significantly higher than that in non-dysphagic cases [39/66 (59.1%)]. However, owing to many missing MSA values, we could not comprehensively analyze the relationship between dysphagia and MSA. Finally, A detailed investigation of the role of swallowing therapy including the type of therapeutic interventions was not clarified.

## Conclusions

In conclusion, almost all IIM patients with dysphagia improved dysphagia severity and the complete oral intake rate within one year was 72.2%. Particularly, almost all non-IBM patients with dysphagia recovered their swallowing functions compared to those with IBM. However, the survival rate in IIM with dysphagia was very poor compared with those without dysphagia, and the overall mortality rate was 44.4%. In addition, malignancy-associated myositis was a poor prognostic factor in patients with IIM and the survival outcomes in IIM patients with dysphagia were poor compared with those without dysphagia when malignancy occurred. Early cancer treatment is crucial in IIM patients with IIM when malignancies occur.
